# From night-to-night and from person-to-person: dynamic phenomena of insomnia

**DOI:** 10.1093/sleepadvances/zpag035

**Published:** 2026-03-23

**Authors:** Mona M Klau, Jaap Lancee, Mathilde I Looman, Han L J Van Der Maas, Renée M Visser, Tessa F Blanken

**Affiliations:** Department of Clinical Psychology, University of Amsterdam, Amsterdam, the Netherlands; Department of Clinical Psychology, University of Amsterdam, Amsterdam, the Netherlands; Department of Clinical Psychology, University of Amsterdam, Amsterdam, the Netherlands; Department of Psychological Methods, University of Amsterdam, Amsterdam, the Netherlands; Department of Clinical Psychology, University of Amsterdam, Amsterdam, the Netherlands; Department of Clinical Psychology, University of Amsterdam, Amsterdam, the Netherlands; Department of Psychological Methods, University of Amsterdam, Amsterdam, the Netherlands

**Keywords:** insomnia, phenomena, dynamics, individual-level analysis

## Abstract

**Study Objectives:**

Sleep is complex and variable, yet insomnia research and treatment often rely on averages—either across nights or across individuals. Such approaches risk obscuring dynamic features that characterize insomnia as a disorder and its unique manifestation in individuals. In this study, we explore disorder-specific (group-level) and person-specific (individual-level) dynamic phenomena of insomnia among people with insomnia.

**Methods:**

We analyzed 8 weeks of sleep diary data from 61 participants with insomnia. Four domains of sleep dynamics were examined at group- and individual-levels: (1) night-to-night variability, (2) temporal dependency of sleep quality, (3) stability of sleep complaints, (4) weekday-weekend variability. We correlated these domains with insomnia severity, pre-sleep arousal, and sleep-related safety behavior.

**Results:**

At the group-level, insomnia was characterized by (1) night-to-night fluctuations in sleep parameters, (2) unpredictable sleep quality, (3) frequent co-occurrence and fluctuations in type of sleep complaints, and (4) different sleep patterns on weekdays and weekends. These disorder-specific dynamic phenomena showed medium-sized significant correlations with insomnia indices, ranging from *r* = –0.25 to 0.41. At the individual-level, all four domains varied markedly across individuals. While the group-level characterizations were fitting for some participants, others showed patterns clearly distinct. We developed a Shiny application which allows readers to explore individual sleep profiles (https://uvasobe.shinyapps.io/PersonalSleepExplorer/).

**Conclusions:**

Sleep in insomnia varies from night-to-night and person-to-person. Reliance on averages across nights and across individuals may obscure fluctuations of potential clinical relevance. We call for broader use of sleep diaries to capture dynamic patterns of insomnia and for investigation of their clinical utility.

**Clinical Trial:**

Sleep Restriction Treatment for Insomnia.

URL: https://clinicaltrials.gov/study/NCT05548907

**Registration:**

NCT05548907.

Statement of Significance Insomnia is typically studied through averaged sleep measures over time and people, yet these may obscure the dynamic and heterogeneous nature of the disorder. This study characterizes how sleep varies from night-to-night and person-to-person, revealing disorder-specific and person-specific patterns of fluctuation. By identifying these dynamic phenomena at both the group and individual level, this work narrows the disconnect between the level at which insomnia is explained, experienced, and treated (processes evolving in individuals across nights) and is usually researched (processes reduced to static group findings). These insights call for consolidation of disorder- and person-specific sleep patterns and for exploration of their clinical utility.

## Introduction

Over the past decades, substantial progress has been made in understanding and treating insomnia [1, 2]. Various authors have provided excellent models and theoretical frameworks that have shaped both our knowledge of the disorder and its treatment [3–7]. These efforts have been instrumental in shaping the current gold standard treatment, Cognitive Behavioral Therapy for Insomnia (CBT-I) [8, 9]. However, up to 50% of patients still do not fully benefit from CBT-I [10]. To increase remission rates, treatment approaches must be refined. Achieving this requires deepening our understanding of insomnia and exploring perspectives that may have been overlooked until now.

One such perspective concerns the dynamic nature of sleep in insomnia. We propose that a better characterization of insomnia in terms of its temporal fluctuations may be crucial to our understanding of the disorder and ultimately lead to further improvements in insomnia treatment. In this paper, we examine the *dynamic nature of sleep in insomnia* from two perspectives. First, by exploring **disorder-specific dynamic phenomena**: robust patterns of fluctuations in sleep across people with insomnia (characterizations at the group-level). Second, by exploring **person-specific dynamic phenomena**: robust patterns of fluctuations in sleep in a specific person with insomnia (characterizations at the individual-level).

**Figure 1:**
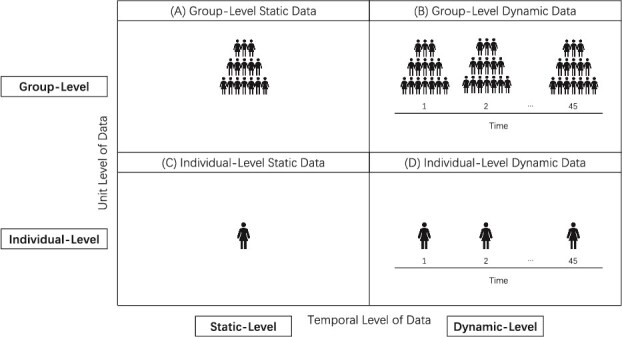
Overview of unit and temporal data levels. Group-level dynamic data (top-right) enables us to establish disorder-specific dynamic phenomena of insomnia. Individual-level dynamic data (bottom-right) enables us to establish person-specific dynamic phenomena of insomnia.

Regarding the first aspect—the fluctuations in sleep across people with insomnia (disorder-specific dynamic phenomena)—it is already recognized that sleep is inherently complex and variable [11]. It fluctuates across the lifespan as well as from night-to-night [12, 13]. Despite the acknowledged importance of variability of sleep, most insomnia research relies on static data providing single time-point measures or values averaged across nights (see Figure 1  static-level) [14]. In the context of insomnia, only a small number of studies to date have focused on identifying dynamic characterizations across people with insomnia. Thus far, it is suggested that individuals with insomnia do experience fluctuations from night-to-night in sleep parameters such as time asleep [12, 15, 16], but there is no consistent evidence for compensatory patterns following poor sleep [17–20]. Furthermore, the dominant sleep complaints (e.g. sleep onset or sleep maintenance problems) can fluctuate over time [21], and sleep timing seems to shift between weekdays and weekends [22, 23]. While these studies represent early contributions to the characterization of disorder-specific dynamic phenomena of insomnia, the literature remains limited in scope, replications are scarce, and these dynamic features have not yet been directly examined in relation to established characterizations such as insomnia severity.

For the second aspect—the fluctuations in sleep within an individual person with insomnia (person-specific dynamic phenomena)—it is crucial to recognize that insomnia is highly heterogeneous [24]. Insomnia is marked by person-to-person differences in sleep-related traits [24, 25] and treatment responses [26]. Despite the acknowledged heterogeneity of insomnia, most insomnia research relies on group data: values averaged across individuals (e.g. reporting mean values estimated across numerous people; see Figure 1A  group-level), which may mask important information about the individual manifestation of insomnia. In addition, while models of insomnia are often formulated at the level of processes evolving in individuals across nights (e.g. an individual going to bed earlier in response to experiencing sleep problems) [5], empirical findings at the individual-level that capture this dynamic aspect are lacking. As a result, there is currently a disconnect between the level at which insomnia is researched (group-level, static) and at which it is explained, experienced, and treated (individual-level, dynamic). Systematic investigations on identifying person-specific dynamic phenomena of insomnia remain largely absent.

That the dynamic phenomena of insomnia—both disorder-specific and person-specific—have often been overlooked is surprising, given that they can be examined with one of the most widely used tools in both clinical and research settings: the sleep diary [27]. Sleep diaries provide night-to-night information on subjective sleep metrics ranging from bedtime to total sleep time, making them well-suited to capture clinically relevant fluctuating patterns at both the group- and individual-levels [28]. Despite patients frequently completing diaries throughout treatment and research studies, these data are rarely analyzed beyond informing treatment.

In this study, we want to address the knowledge gap by utilizing sleep diaries to identify which dynamic features of sleep characterize insomnia as a disorder and which fluctuations may characterize a specific person with insomnia. Given the early stages of research on sleep dynamics in insomnia, it is still unknown which variability measures should best be used to assess insomnia [15]. Accordingly, to make an initial insomnia variability assessment, we select four clinically relevant dynamic domains of sleep that were identified in previous studies and examine them at both group- and individual-levels: (1) night-to-night variability [12, 15, 16]; (2) temporal dependency of sleep quality [17–20]; (3) stability of sleep complaints [21]; (4) weekday-weekend variability [22, 23]. Importantly, we select these domains based on previous research, but we do not consider this a final or exhaustive set of dimensions by which to characterize the dynamics of sleep. Additionally, because it remains largely unknown whether variability-based characterizations capture aspects of insomnia that are distinct from more established measures, we explore to what extent these novel, dynamic characterizations of insomnia overlap with indices of insomnia severity, pre-sleep arousal, and sleep-related safety behavior. By investigating these dynamic characterizations, we hope not only to refine our conceptual understanding of insomnia, but also to generate insights with potential implications for treatment approaches.

## Materials and Methods

### Participants

This was a secondary analysis of a previously published trial on the efficacy of sleep restriction therapy [29]. In this paper we report on the control condition who only completed the sleep diaries (*n* = 61). Participants were recruited via online advertisements (www.insomnie.nl) and social media platforms (Instagram and Facebook). Eligibility criteria included (1) an Insomnia Severity Index (ISI) score ≥ 10, (2) self-reported insomnia complaints of ≥30 minutes of wakefulness per night, ≥3 nights per week, for ≥3 months, with associated daytime impairment, and (3) self-reported sleep efficiency <85% based on a 7-day baseline sleep diary. Participants had to be Dutch-speaking adults (≥18 years). Exclusion criteria were: (1) recent CBT-I (within 12 months), (2) recent initiation of psychological treatment (within 6 months), (3) recent psychoactive medication changes (within 6 weeks), (4) diagnoses of psychosis or schizophrenia, (5) severe depressive symptoms (BDI-II ≥29) or active suicidal ideation (BDI-II item 9 ≥ 2), (6) pregnancy or breastfeeding, (7) night shift work, or (8) no internet access. Use of sleep medication was permitted.

### Procedure

All data were collected online through Qualtrics (https://www.qualtrics.com). Following informed consent and initial screening, participants completed 8 weeks of online sleep diaries and received automated weekly feedback on sleep diary averages. At the beginning of every week, participants additionally completed assessments of insomnia severity, pre-sleep arousal, and sleep-related safety behavior. Our current sample did not receive any behavioral or therapeutic sleep intervention. The trial was registered in clinicaltrials.org (NCT05548907) and was approved by the internal ethical review committee of the University of Amsterdam. See the parent trial [29] for full description of the procedures during the study period.

### Measures

#### Sleep diary

Time in bed (TIB, min), total sleep time (TST, min), bedtime (BT, timestamp), risetime (RT, timestamp), sleep onset latency (SOL, min), wake time after sleep onset (WASO, min), and sleep efficiency (SE, %; $\frac{\mathrm{TST}}{\mathrm{TIB}}\times 100$) were measured by questions from the Consensus Sleep Diary (CSD) [27].

#### Insomnia severity, pre-sleep arousal, and sleep-related safety behavior

Insomnia severity was measured with the Insomnia Severity Index (ISI) [30]. The ISI is a 7-item self-report questionnaire on sleep difficulties (sleep onset, sleep maintenance, early morning awakenings), and sleep difficulty consequences (sleep dissatisfaction, interference with daily functioning, noticeability of sleep problems by others, and distress caused by sleep difficulties). Each item is rated on a 5-point Likert scale (0-4), yielding a total score ranging from 0 to 28. Higher scores reflect more severe insomnia. The ISI has demonstrated good internal consistency and validity as a screener and outcome measure [30, 31].

Pre-sleep arousal was measured with the Pre-Sleep Arousal Scale (PSAS), a 16-item questionnaire rated on a 5-point Likert scale (1-5), yielding total scores ranging from 16 to 80 [32]. Higher scores reflect greater levels of pre-sleep arousal. An example item is: “Being mentally alert, active”. The PSAS has demonstrated good internal reliability and validity [33].

Sleep-related safety behavior was measured with the Sleep-Related Behaviors Questionnaire (SRBQ), a 32-item self-report measure [34]. Each item is rated on a 5-point Likert scale (0-4), yielding a total score ranging from 0 to 128. Higher scores indicate greater use of behaviors intended to prevent poor sleep. An example item is: “I take on fewer social commitments”. The SRBQ has demonstrated good internal reliability and validity [34].

### Statistical analyses

To investigate dynamic phenomena of insomnia, we looked at four dynamic domains of sleep at both group- and individual-levels:

(1) We assessed the *night-to-night variability* of sleep metrices to see how much TIB, TST, BT, RT, SOL, WASO, and SE fluctuate from night-to-night. To quantify this variability, we calculated the within-person standard deviation for each parameter (average change from own mean).
(2) We examined the *temporal dependency of sleep quality*, assessing whether good and bad nights follow predictable patterns. We calculated the lag-1 autocorrelation of SE to see whether the quality of one night provides predictive value for the next. Autocorrelation coefficients are bounded between –1 and 1. Values near 1 indicate strong positive dependence (high sleep quality night followed by another high sleep quality night, and low by low), values near –1 indicate strong negative dependence (high sleep quality night followed by low sleep quality night, and vice versa), and values near 0 indicate unpredictability (no consistent temporal structure between consecutive nights). We further categorized nights into good, neutral, and bad nights, based on each individual’s personal distribution of SE values: the highest tertile as “good nights,” the middle tertile as “neutral nights,” and the lowest tertile as “bad nights.” Using these categories, we constructed a transition matrix summarizing the probabilities of moving from one category to another on consecutive nights.
(3) We investigated the *stability of sleep complaints*, examining whether sleep complaints tend to occur independently (difficulty falling asleep vs. difficulty staying asleep) or co-occur in one night and whether the complaint type fluctuates across nights or stays stable. To assess the complaint type per night, we categorized each night based on the presence of prolonged SOL (>30 minutes) and/or prolonged WASO (>30 minutes). Nights were classified into four types: (1) prolonged SOL only, (2) prolonged WASO only, (3) both prolonged SOL and WASO, or (4) none. We then calculated the percentage of nights per type. To examine the stability of complaint types across nights, we calculated Shannon entropy based on the proportion of nights with each type to quantify the variability in complaint type, with higher entropy reflecting greater fluctuation (0 = complete consistency in type; 2 = maximum variability across all types).
(4) We looked at the *weekday-weekend variability* to explore whether sleep varies depending on the type of day (weekday or weekend). To quantify this variability, we computed the absolute difference score between weekday nights (Sun-Mon, Mon-Tue, Tue-Wed, Wed-Thu, Thu-Fri) and weekend nights (Fri-Sat, Sat-Sun) for TIB, TST, BT, RT, SOL, WASO, and SE.

For our first aim, to establish disorder-specific dynamic phenomena of insomnia, we analyzed these four domains at the group-level. To evaluate their relations with established characterizations, we conducted zero-order correlations between the dynamic characterizations (night-to-night variabilities, SE lag-1 autocorrelation, Shannon entropy, weekday-weekend variabilities) and insomnia severity, pre-sleep arousal, and sleep-related safety behavior. Because night-to-night variability may partly reflect differences in mean levels, we conducted follow-up partial correlations for this domain to test whether variability was associated with outcomes over and above mean sleep values (e.g. night-to-night variability of SOL and ISI while controlling for mean SOL). For our second aim, to establish person-specific dynamic phenomena, we assessed these four domains at the individual-level.

## Results

### Sample characteristics

The sample consisted of 49 women (80.3%) and 12 men (19.7%) with a mean age of 46 years (SD = 14). On average, participants spent 8 hours and 14 minutes in bed per night (SD = 1h20m) between 23:30 (SD = 1h19m) and 07:44 (SD = 1h23m). Of the time in bed, they reported an average of 5 hours and 56 minutes of total sleep time (SD = 1h44m), resulting in a mean sleep efficiency of 72.6% (median = 76.7%; SD = 19.2%). On average, sleep onset latency was 55 minutes (SD = 1h7m), while wakefulness after sleep onset was 1 hour and 28 minutes (SD = 1h19m). The mean insomnia severity experienced by our sample was 17.30 (SD = 4.80), mean pre-sleep arousal was 38.68 (SD = 10.50), and mean sleep-related safety behavior was 42.80 (SD = 19.61).

### Disorder-specific dynamic phenomena (group-level)

To identify disorder-specific dynamic insomnia—robust patterns of fluctuations in sleep across people with insomnia—we examined the four dynamic domains at the group-level (see Figure 1B) and their relations to well-known characterizations of insomnia.

#### Night-to-night variability

**Figure 2:**
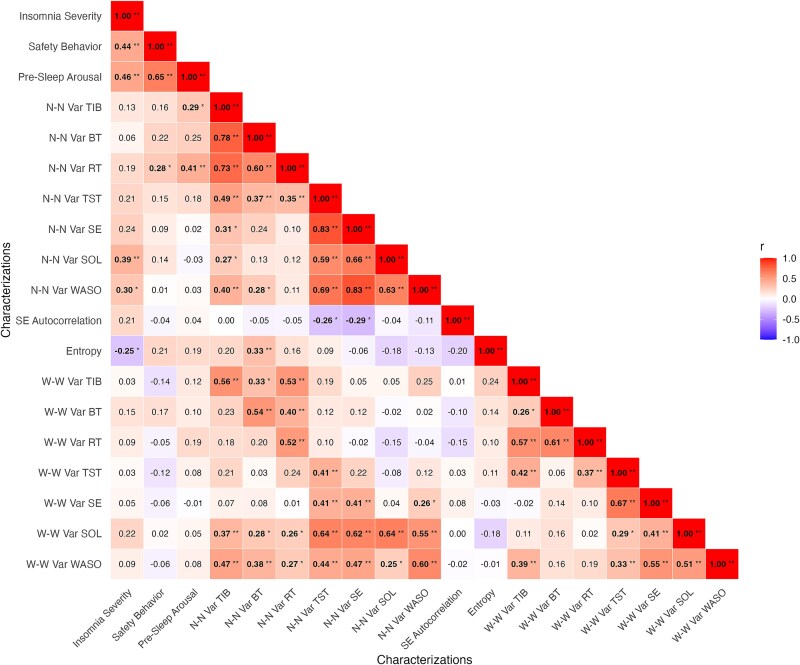
Visualization of zero-order correlations between dynamic and well-known characterizations of insomnia. *p*<.05 (*); *p*<.01 (**). N-N Var = night-to-night variability. W-W Var = weekday-weekend variability. Bold values are significant correlations.

We assessed to what extent sleep parameters fluctuated from night-to-night. Across the sample, participants varied on average 1 hour and 3 minutes (SD = 23m) in terms of how much time they spent in bed per night, with bedtime and risetime showing night-to-night variability of 55 minutes (SD = 25m) and 56 minutes (SD = 22m), respectively. Participants also varied in terms of how much they slept per night, by an average of 1 hour and 15 minutes (SD = 21m). The average night-to-night variability of sleep onset latency was 39 minutes (SD = 28m) and wake after sleep onset varied by 54 minutes (SD = 28m). These nightly fluctuations in sleep timing and continuity corresponded to a group-level average night-to-night variability in SE of 12.4% (SD = 4.4%). Night-to-night variability measures showed various significant small to medium zero-order correlations (*r* = 0.28 – 0.41) with insomnia severity, pre-sleep arousal, and safety behavior (see Figure 2). When accounting for sleep values, we observed significant partial correlations between pre-sleep arousal and night-to-night variability of time in bed (*r_p_* = 0.30) as well as night-to-night variability of risetime (*r_p_* = 0.40) and between safety behaviors and night-to-night variability of risetime (*r_p_* = 0.27). These partial correlations can be interpreted as, for example, that greater night-to-night fluctuations in time in bed were associated with higher pre-sleep arousal, over and above the amount of time spent in bed.

#### Temporal dependency of sleep quality

We examined to what extent poor sleep may be followed by compensatory improvements. The group data provided little evidence for this: sleep efficiency demonstrated minimal night-to-night consistency, with a lag-1 autocorrelation of only 0.04 (SD = 0.14). Thus, the quality of one night’s sleep offered almost no predictive value for the next. Subsequently, we examined this unpredictable pattern by computing the probability of transitioning between bad, neutral, or good nights. At the group level, nights that followed a bad night were approximately equally likely to be bad (0.35, SD = 0.09), neutral (0.33, SD = 0.10), or good (0.31, SD = 0.11), offering no evidence of a compensatory pattern (see bold row in Table 1). The lag-1 autocorrelation of sleep efficiency showed a small to medium zero-order correlation with insomnia severity (*r* = 0.21), however this association was not significant. Zero-order correlations with pre-sleep arousal and safety behavior were near zero (see Figure 2).

**Table 1:** Group Transition Matrix between Bad, Neutral, and Good Nights

	Bad night M (SD)	Neutral night M (SD)	Good night M (SD)
Bad night	**0.35 (0.09)**	**0.33 (0.10)**	**0.31 (0.11)**
Neutral night	0.37 (0.09)	0.35 (0.11)	0.29 (0.10)
Good night	0.32 (0.10)	0.33 (0.12)	0.35 (0.11)

Missing nights were assumed to be missing at random and were therefore excluded from analysis. The table presents the group *transition matrix* showing the average probability of moving from one sleep quality (rows) to another (columns) on consecutive nights. For example, the bolded row illustrates the probabilities of experiencing a bad, neutral, or good night following a bad night. The diagonal values represent the likelihood that the same sleep quality recurs on the next night, reflecting temporal stability. Higher diagonal values indicate greater night-to-night consistency, whereas higher off-diagonal values indicate more frequent changes in sleep quality. Values around 0.33 suggest random transitions, where each sleep quality is equally likely regardless of the previous night.

#### Stability of sleep complaints

**Figure 3:**
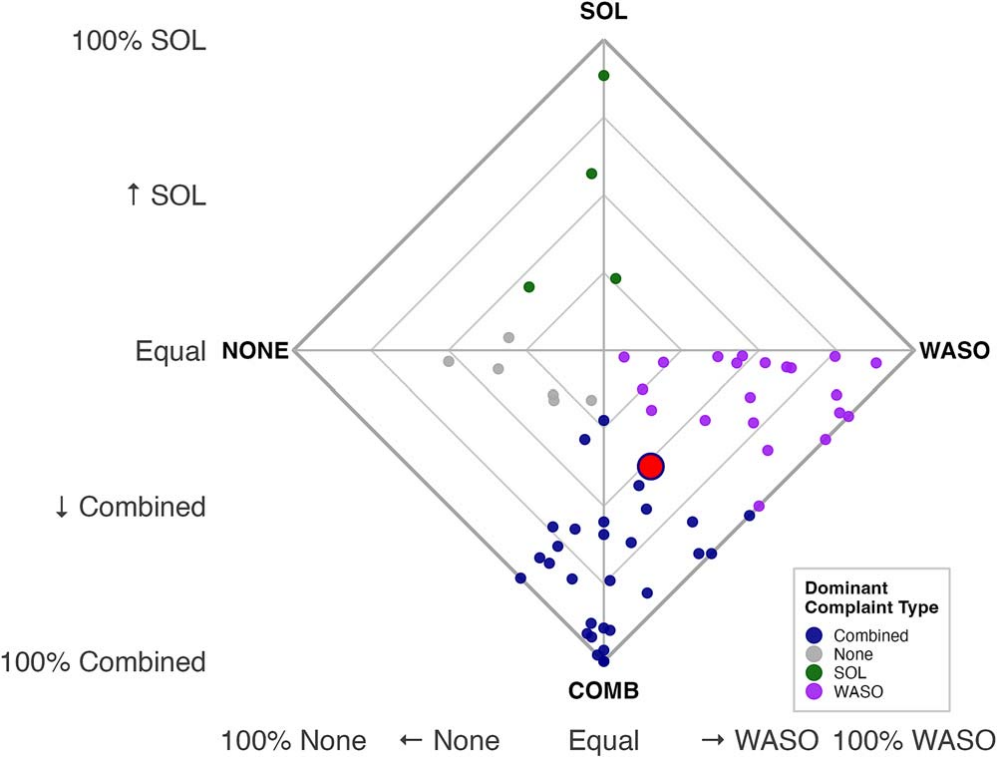
Visualization of dominant sleep complaints. Each dot represents an individual and indicates their dominant complaint type, defined as the complaint occurring on the largest proportion of nights. For example, the point located near the top of the plot (green) represents an individual for whom SOL is the dominant complaint (i.e., nearly all nights involve SOL-only). The larger dot (red) represents the group average, indicating that, on average, combined complaints (SOL and WASO) occur on 47% of nights, WASO-only on 30%, SOL-only on 9%, and no complaints on 15% of nights.

We examined to what extent sleep complaints co-occur within one night and their consistency across nights. We saw that across participants, the co-occurrence of difficulties falling asleep (SOL) and staying asleep (WASO) was the most common pattern and experienced on average almost half of the nights (M = 46.5%; SD = 32.8%), followed by maintenance-only (WASO) complaints (M = 29.7%, SD = 29.0%) and onset-only (SOL) complaints (M = 9.3%, SD = 17.4%). Nights without any complaint were also present (M = 14.5%, SD = 15.7%). These proportions indicate that, at the group-level, individuals more frequently experienced both complaints concurrently in a single night, rather than isolated difficulty with either falling or staying asleep (see red dot in Figure 3).

We also found that at the group-level, complaint types across nights were moderately variable. The average entropy was 1.08 (SD = 0.52), indicating that participants did not consistently experience the same complaint type every night (entropy = 0), nor did they fluctuate randomly (entropy = 2). This suggests a moderate level of instability in how sleep complaints presented across nights. Entropy of sleep complaints showed small to medium zero-order correlations with insomnia severity, pre-sleep arousal, and safety behavior (see Figure 2). However, only the relation to insomnia severity was significant (*r* = –0.25), meaning that the more stable the complaint type, the higher the insomnia severity experienced.

#### Weekday-weekend variability

We examined to what extent people with insomnia varied in their sleep when comparing weekdays (Sunday through Thursday nights) to weekends (Friday and Saturday nights). On average, from weekdays to weekend, time in bed varied by 29 minutes (SD = 24m), bedtime by 35 minutes (SD = 31m), and risetime by 45 minutes (SD = 36m). Total sleep time varied by 28 minutes (SD = 26m), and sleep efficiency by 3.6% (SD = 3.9%) from weekdays to weekends. Variability between weekdays and weekends was also observed in sleep onset latency (M = 12m, SD = 12m) and wake after sleep onset (M = 20m, SD = 19m). For most sleep parameters, night-to-night variability exceeded weekday-weekend variability, indicating that weekday-weekend effects may not fully account for the observed fluctuations in sleep (see Supplement Table S1). Consistent with this, correlations between night-to-night and weekday-weekend variability within the same parameter were below 1, suggesting these metrics capture only partly overlapping sources of variation (see Figure 2). Zero-order correlations between weekday-weekend variability measures and insomnia severity, pre-sleep arousal, or safety behavior were small and did not reach statistical significance (see Figure 2).

### Person-specific dynamic phenomena (individual-level)

While the group-level dynamic findings provide important insight into the dynamic patterns that may characterize insomnia as a disorder (see Figure 1B), all four novel characterizations revealed large standard deviations indicating marked heterogeneity across individuals (see standard deviations and Supplement Figures S1–S3). Consequently, disorder-specific dynamic phenomena may obscure distinct person-specific dynamic phenomena that emerge only when examining insomnia at the individual-level (see Figure 1D).

To identify person-specific dynamic insomnia—robust patterns of fluctuations in sleep within a specific person with insomnia—we examined the four dynamic domains at the individual-level for specific individuals (see Figure 1D). Dynamic sleep profiles for each individual participant can be found and explored in this Shiny Application (https://uvasobe.shinyapps.io/PersonalSleepExplorer/). Here, we highlight two illustrative cases, Emma and Sarah (fictious names), to demonstrate how dynamic phenomena can manifest differently for a specific person with insomnia (i.e. at the individual-level), supporting the need for person-centered approaches to understand and treat insomnia.

#### Emma—The highly variable sleeper with no stable complaint type (No. 44)

**Figure 4:**
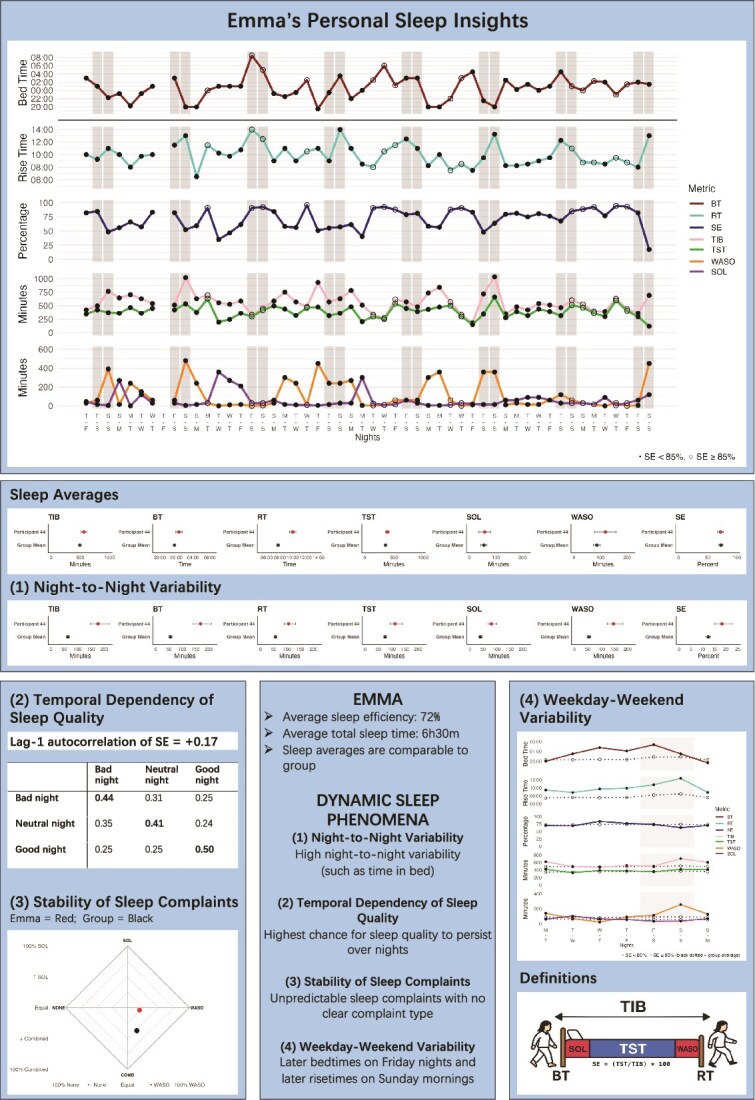
Emma’s personal sleep overview.

Emma’s profile showed marked **night-to-night variability** for multiple sleep parameters. Time in bed, bedtime, and wake after sleep onset each fluctuated by more than two hours across the nights, despite her sleep averages being comparable to group-level findings (explore on Shiny App Sleep Averages vs. Night-to-Night Variability). This illustrates that simply interpreting the overall sleep metrices averaged across nights (see Figure 1C) can obscure more fine-grained patterns that are likely of importance in treatment. In terms of **temporal dependency of sleep quality**, for Emma, poor nights tended to cluster together, with a positive lag-1 autocorrelation of sleep efficiency (*r* = 0.17). If she experienced a bad night, the chances were highest that the next night would also be bad. Her **stability of sleep complaints** was low: 27% of nights involved prolonged sleep onset, 31% prolonged wake after sleep onset, and 31% both. This yielded a very high entropy score (entropy = 1.92), reflecting high unpredictability of the complaint type Emma experienced. Finally, she also exhibited **weekday-weekend variability** for several sleep parameters, including time in bed (2h4m difference), bedtime (1h10m difference), and wake after sleep onset (1h54m difference). Sleep efficiency remained more stable across the week (5.8% difference). For an overview of Emma’s sleep, see Figure 4.

#### Sarah—The compensatory sleeper with combined sleep complaints (No. 53)

Sarah presented a sleep profile with sleep averages and **night-to-night variability** that were similar to group-level findings. Regarding the **temporal dependency of sleep quality**, she showed a compensatory dynamic, with a negative lag-1 autocorrelation of sleep efficiency (*r* = –0.23), indicating that for Sarah quality of sleep tends to oscillate. In fact, after a bad night, Sarah was most likely to experience a good night. Her **stability of sleep complaints** was characterized by prominence of combined difficulties: 76% of nights involved both prolonged sleep onset latency and wake after sleep onset, while isolated complaints were relatively rare. Relatedly, her entropy score was moderate (entropy = 1.11), indicating that combined complaints were most frequent, though some fluctuation across complaint types remained. Finally, Sarah also exhibited **weekday-weekend variability** in several sleep parameters, particularly total sleep time (47m difference) and risetime (1h40m difference). Time in bed, sleep onset latency, wake after sleep onset, and sleep efficiency varied less across the week (29m, 4m, 22m, and 4.1% difference, respectively).

### The importance of individual-level data

These two specific individuals with insomnia illustrate how insomnia can manifest across nights differently per person, something we cannot unravel when only looking at data averaged across individuals (group-level data) or averaged across nights (static-level data). Emma’s insomnia was characterized by high night-to-night variability, persisting sleep quality, and unpredictability in complaint type with weekday-weekend fluctuations particularly regarding difficulties maintaining sleep. Sarah’s insomnia, in contrast, was characterized by less night-to-night and weekday-weekend variability, compensatory dynamics of sleep quality, and a combined complaint type. Emma’s and Sarah’s person-specific dynamic phenomena differed from disorder-specific dynamic phenomena of insomnia in various domains. Together, they demonstrate how distinct dynamic sleep patterns can emerge at the individual-level (see Figure 1D), beyond what group-level insights can reveal (see Figure 1B).

## Discussion

In this study, we aimed to advance our understanding of insomnia by characterizing its dynamic nature. To do so, our goal was to establish disorder-specific dynamic phenomena—robust patterns of fluctuations in sleep across people with insomnia (group-level), as well as person-specific dynamic phenomena—robust patterns of fluctuations in sleep in a specific person with insomnia (individual-level). We examined four clinically relevant domains of sleep dynamics at both the group-level and the individual-level: (1) night-to-night variability, (2) temporal dependency of sleep quality, (3) stability of sleep complaints, and (4) weekday-weekend variability.

The most interesting finding emerged when comparing the dynamic phenomena at the group- and individual-levels. For example, at the group-level, fluctuations in sleep parameters reached up to 1 hour and 15 minutes which is in line with previous research which emphasized the variable nature of sleep in insomnia [12, 15, 16]. However, when looking at person-specific fluctuations, we saw considerable heterogeneity from person-to-person. For some individuals, night-to-night fluctuations in sleep were almost absent, while for another they spanned up to 3 hours (e.g. Emma).

A similar observation was made for the temporal dependency of sleep quality. The way in which quality of sleep fluctuated appeared largely unpredictable, providing little evidence for disorder-compensatory dynamics. In contrast, person-specific profiles revealed distinct temporal structures. Some individuals showed clustering, where bad nights tended to follow by additional bad nights (e.g. Emma), whereas others displayed compensatory dynamics, where bad nights were most often followed by good nights (e.g. Sarah). Our findings align with previous reports showing no predictable pattern in night-to-night variations of sleep quality at the group-level but predictable patterns for some individuals [17–20]. For a discussion on potential subgroups, we refer to [17, 18].

This heterogeneity also extended to the stability of sleep complaints. Group-level analyses suggested that problems with falling asleep and staying asleep tended to co-occur within nights, with moderate fluctuations in complaint type across nights. This finding underlines the instability of complaint types and adds to previous research showing fluctuations in sleep complaints and types over time [21]. However, individual profiles again differed from person-to-person: some participants showed a stable dominant complaint, while others experienced shifts in complaint type across nights (e.g. Emma).

Finally, a comparable observation emerged for weekday-weekend variability. For example, at the group-level, variability in bedtime between weekdays and weekends was 35 minutes, which is in line with previous reports [22]. Yet, when examined at the individual-level, some individuals maintained highly consistent sleep patterns across the week, whereas others showed variations of 2 hours and 37 minutes in bedtime.

We examined to what extent these dynamic characterizations of insomnia represent novel additions to more well-known characterizations of insomnia, including insomnia severity, pre-sleep arousal, and sleep-related safety behavior. While some modest relations were present, many correlations were weak or nonsignificant. This suggests that while dynamic phenomena show some overlap with established measures, they also capture distinct aspects of insomnia that are not reflected in conventional characterizations.

While this study underscores the importance of investigating sleep dynamically, several limitations should be considered. First, although the four dynamic characterizations of insomnia examined in this study were selected based on prior research and clinical relevance, this choice was necessarily selective. At present, there are no established guidelines or consensus regarding which variability measures should be used to assess insomnia [15], reflecting the early stage of research on sleep dynamics. Consequently, other potentially informative variability metrics or dynamic features may exist that were not captured by our approach. Our findings should therefore be interpreted as an initial exploration rather than a comprehensive characterization of insomnia’s dynamic features.

Second, the disorder-specific phenomena identified were derived exclusively from individuals with insomnia recruited as the control group of a sleep restriction therapy trial. All participants met self-reported DSM-5 criteria for insomnia. However, in addition a trial-specific inclusion criteria (sleep efficiency <85%) was applied. Moreover, the current study has not yet contrasted our findings with a healthy sample. As a result, it remains unclear how our disorder-specific phenomena compare to dynamics in individuals without insomnia and to what extent our novel characterizations capture aspects of insomnia beyond established characterizations when considered across a broader spectrum of sleepers.

Third, the generalizability of our findings is still unknown. Participants were Dutch-speaking and predominantly female (80.4%). This relative demographic and cultural homogeneity may limit the extent to which the identified dynamic phenomena generalize to populations with different racial, ethnic, cultural, or sex/gender distributions**.** In addition, in more heterogeneous samples the variability from person-to-person may be even bigger than we demonstrated in our current study.

A final limitation concerns that while we believe that these dynamic characterizations have the potential to inform treatment by providing fine-grained insights into sleep fluctuations that characterize both insomnia as a disorder and an individual patient; however, their clinical utility remains an open question. Future research is needed to determine whether these dynamic features can guide treatment personalization (Shiny Application; Figure 4) or predict treatment response.

We demonstrated that in people suffering from insomnia, sleep varies considerably from night-to-night and from person-to-person. Therefore, overlooking these dynamic patterns may obscure relevant features that are characteristic to the disorder and to the experience of a specific person. It is striking that this dynamic information is in fact collected in most studies on insomnia and during most treatments. We therefore call for:

(1) **Greater utilization of sleep diary data:** Sleep diaries are already widely collected in research and clinical practice, yet their potential is rarely realized beyond insights averaged across nights or pre-post comparisons. Analyzing diaries dynamically, by considering its fluctuations across nights, allows researchers to uncover fine-grained processes that cannot be seen otherwise and enables clinicians to identify intervention targets and track changes in patients’ sleep patterns. Research is needed to look into how dynamic characterizations relate to treatment effects and their utility as intervention targets in treatment.
(2) **Consolidation of disorder-specific dynamic phenomena:** Our findings suggest that insomnia is characterized by disorder-specific dynamic phenomena, such as night-to-night variability in sleep parameters, unpredictable sleep quality, and combined sleep complaints. Research is needed to replicate, validate, and extend these findings in more (diverse) samples including comparisons with individuals without insomnia. Establishing such robust disorder-specific dynamic patterns is essential for building formal models of insomnia, while in clinical practice these insights can guide expectations of how insomnia typically unfolds.
(3) **Systematic investigation of person-specific dynamic phenomena:** Dynamic features of insomnia are heterogeneous across individuals. Utilizing sleep diary data to examine person-specific patterns of sleep across nights, such as seen in our tool (Shiny Application) and Emma’s Sleep Overview (Figure 4) allows researchers to validate models at the same level at which they are formulated and importantly might help clinicians and patients during treatment. Embracing person-specific profiles supports the development of individualized treatment strategies and may improve patient outcomes.

Our findings contribute to the literature on establishing phenomena of insomnia. We showed that across people with insomnia, sleep varied from night-to-night. The degree and pattern of these fluctuations differed widely from person-to-person. This underscores that current common approaches—utilizing values averaged across nights (see Figure 1  static-level) and averaged across individuals (see Figure 1  group-level)—may not adequately capture the dynamic and heterogeneous character of insomnia. We call for additional research on insomnia’s dynamic phenomena across both group- and individual-levels to advance models that reflect the lived experience of insomnia and that may, over time, help to inform treatment.

## Supplementary Material

Supplement_Materials_zpag035.docx

## Data Availability

Informed consent did not include public data sharing. The data underlying this study are thus available upon reasonable request from the corresponding author (MM Klau), but not publicly available.
