# Supplementary material for: From night-to-night and from person-to-person: dynamic phenomena of insomnia
Source: Sleep Adv. 2026 Mar 23;7(2):zpag035. doi: 10.1093/sleepadvances/zpag035 (PMC13140563; doi:10.1093/sleepadvances/zpag035)
Supplement: Supplement_Materials_zpag035.docx [file supplement_materials_zpag035.docx]

**Figure S1**

*Visualization of Night-to-Night Variability*

**

*Note.* Each black point represents an individual participant’s within-person standard deviation (SD) across nights, with horizontal bars showing that participant’s 95% confidence interval. The red point indicates the group mean, with the horizontal bar showing the 95% confidence interval across participants. Higher values indicate higher variability over nights.

**Figure S2**

*Visualization of Weekday-Weekend Variability*

**

*Note.* Each black point represents an individual participant’s absolute difference between weekdays and weekends. The red point indicates the group mean, with the horizontal bar showing the 95% confidence interval across participants. Higher values indicate higher differences in sleep between weekday nights and weekend nights.

**Figure S3**

*Visualization of the Temporal Dependency of Sleep Quality and Stability of Sleep Complaints*

*Note.* Left: Each black point represents an individual participant’s lag-1 autocorrelation of sleep efficiency. The red point indicates the group mean. The dashed line at 0 marks no dependence of sleep quality from one night to the next. Positive values indicate clustering of sleep efficiency from one night to the next. Negative values indicate alternating of sleep efficiency from one night to the next. Right: Each black point represents an individual participant’s Shannon entropy of nightly complaints. The red point indicates the group mean. The dashed line at 1.0 marks moderate fluctuations. Low values indicate higher stability in the type of sleep complaints. High values indicate higher variability in the type of sleep complaints.

**Table S1**

*Comparison of Weekday-Weekend Variability to Night-to-Night Variability*

| Sleep Parameter | Weekday-to-Weekend Variability | Night-to-Night Variability |
| --- | --- | --- |
|  | M (SD) | M (SD) |
| **Time in bed** | 29min (24min) | 1h03min (23min) |
| **Bedtime** | 35min (31min) | 55min (25min) |
| **Risetime** | 45min (36min) | 56min (22min) |
| **Total sleep time** | 28min (26min) | 1h15min (21min) |
| **Sleep efficiency** | 3.6% (3.9%) | 12.4% (4.4%) |
| **Sleep onset latency** | 12min (12min) | 39min (28min) |
| **Wake after sleep onset** | 20min (19min) | 54min (28min) |
